# Supplementary material for: Uncovering the small proteome of Methanosarcina mazei using Ribo-seq and peptidomics under different nitrogen conditions
Source: Nat Commun. 2024 Oct 6;15:8659. doi: 10.1038/s41467-024-53008-8 (PMC11456600; doi:10.1038/s41467-024-53008-8)
Supplement: Supplementary file 1 — Supplementary Information [file 41467_2024_53008_MOESM1_ESM.pdf]

Uncovering the small proteome of *Methanosarcina mazei* using Ribo-seq and peptidomics under different nitrogen conditions

Muhammad Aammar Tufail[1], Britta Jordan[1], Lydia Hadjeras[2], Rick Gelhausen[3], Liam Cassidy[4], Tim Habenicht[1], Miriam Gutt[1], Lisa Hellwig[1], Rolf Backofen[3], Andreas Tholey[4], Cynthia M. Sharma[2], Ruth A. Schmitz[1,#]

[1] Institute for General Microbiology, Kiel University, 24118 Kiel, Germany

[2] Institute of Molecular Infection Biology, University of Würzburg, 97080 Würzburg, Germany

[3] Bioinformatics Group, Department of Computer Science, University of Freiburg, 79110 Freiburg, Germany

[4] Systematic Proteome Research & Bioanalytics, Institute for Experimental Medicine, Kiel University, 24105 Kiel, Germany

[#]: Correspondence: rschmitz@ifam.uni-kiel.de

Keywords:

Archaea; Methanoarchaea; Ribosome profiling; Ribo-seq; small proteome; sORF; nitrogen; dual-function RNAs, proteoform

Supplementary Figures

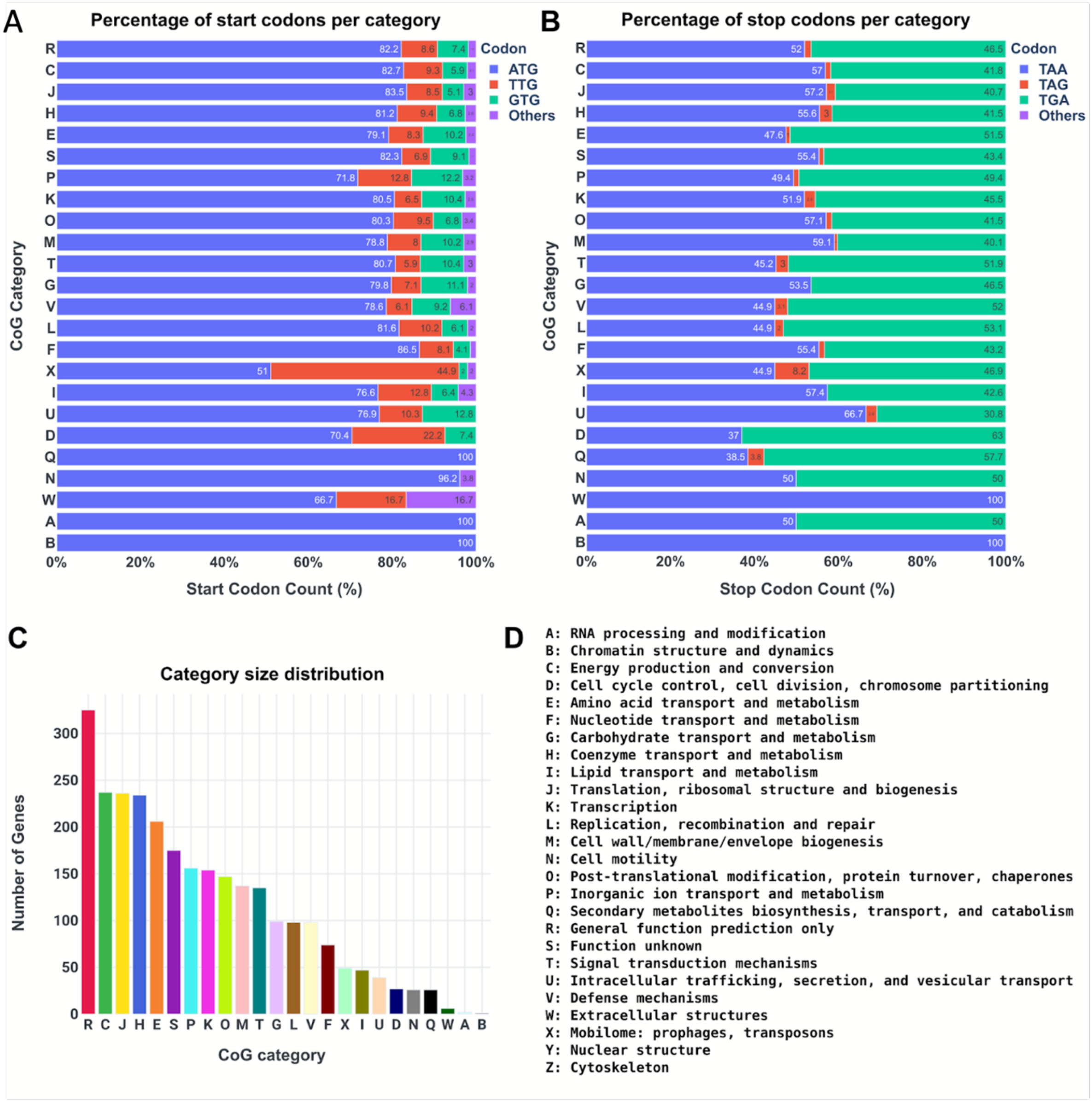

Supplementary Figure 1: Codon Usage Analysis in Clusters of Orthologous Groups (CoGs)

(A) Percentage share of each start codon within CoG categories. **(B)** Percentage share of each stop codon within CoG categories. **(C)** Size distribution of CoG categories, indicating the number of genes in each category. **(D)** Glossary of CoG category symbols with detailed descriptions. The data was obtained by mapping CoG categories from the NCBI database to our annotated genes.

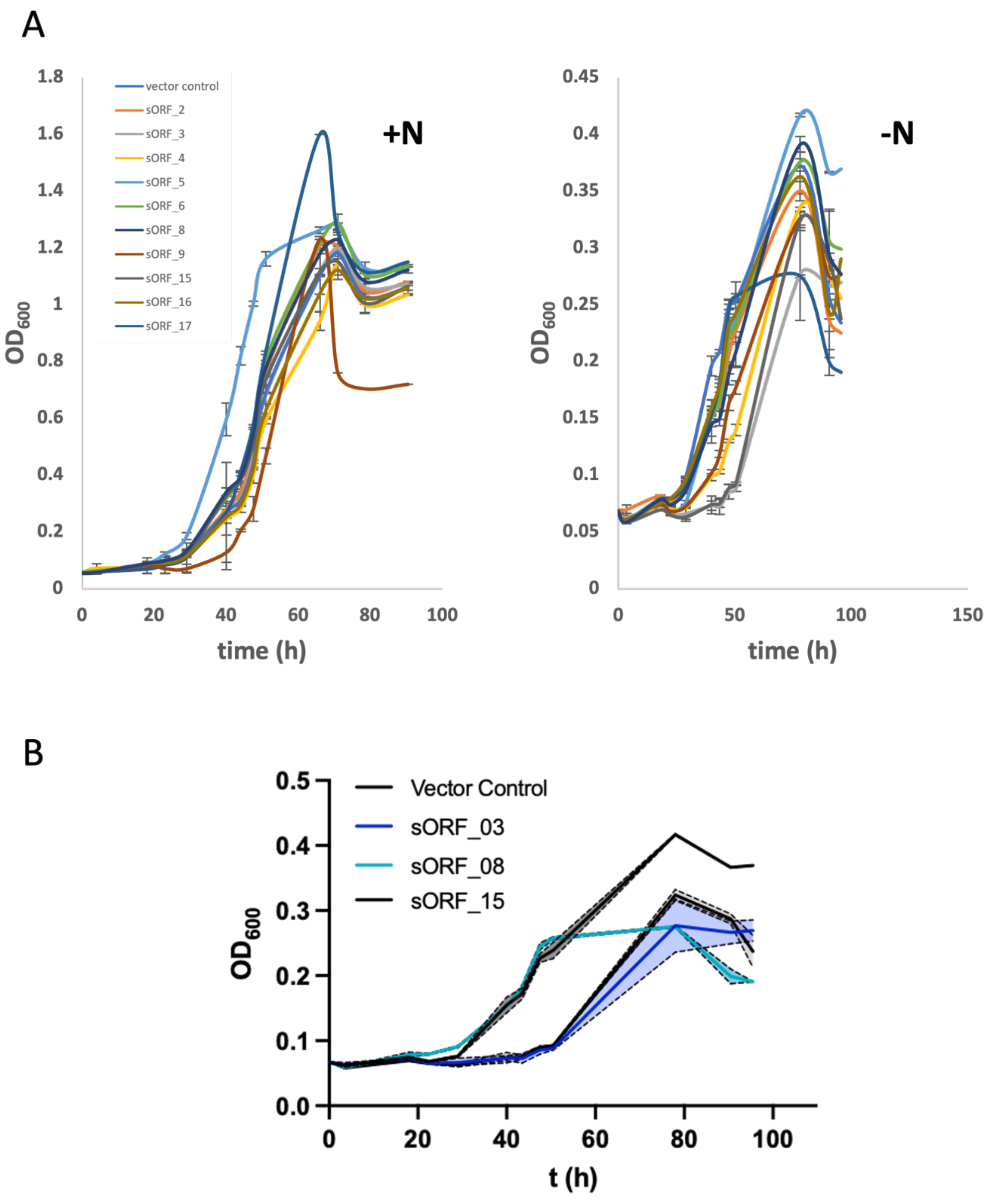

Supplementary Figure 2: Growth analysis of *M. mazei* expressing selected small proteins under their respective native promoter.

(A) Depicted are the growth curves of the *M. mazei* strains expressing selected small proteins under control of their respective native promoter and ribosome binding site under nitrogen sufficiency (+N, left panel) as well as nitrogen limitation (-N, right panel) together with the empty vector control with puromycin (5 µg/ml) as selection marker, each in three biological replicates and standard deviation for

each time point is indicated. The growth was monitored by measuring the optical density at 600 nm (OD_{600}) over the time.

(B) Growth analysis under –N conditions of *M. mazei* expressing sORF_03, sORF_08 and sORF_15 under their respective native promoter in comparison to the empty vector control with puromycin (5 µg/ml) as selection marker, each in three biological replicates and standard deviation for each time point is indicated. The growth was monitored by measuring the optical density at 600 nm (OD_{600}) over the time. Source data for A and B are provided as a Source Data file.

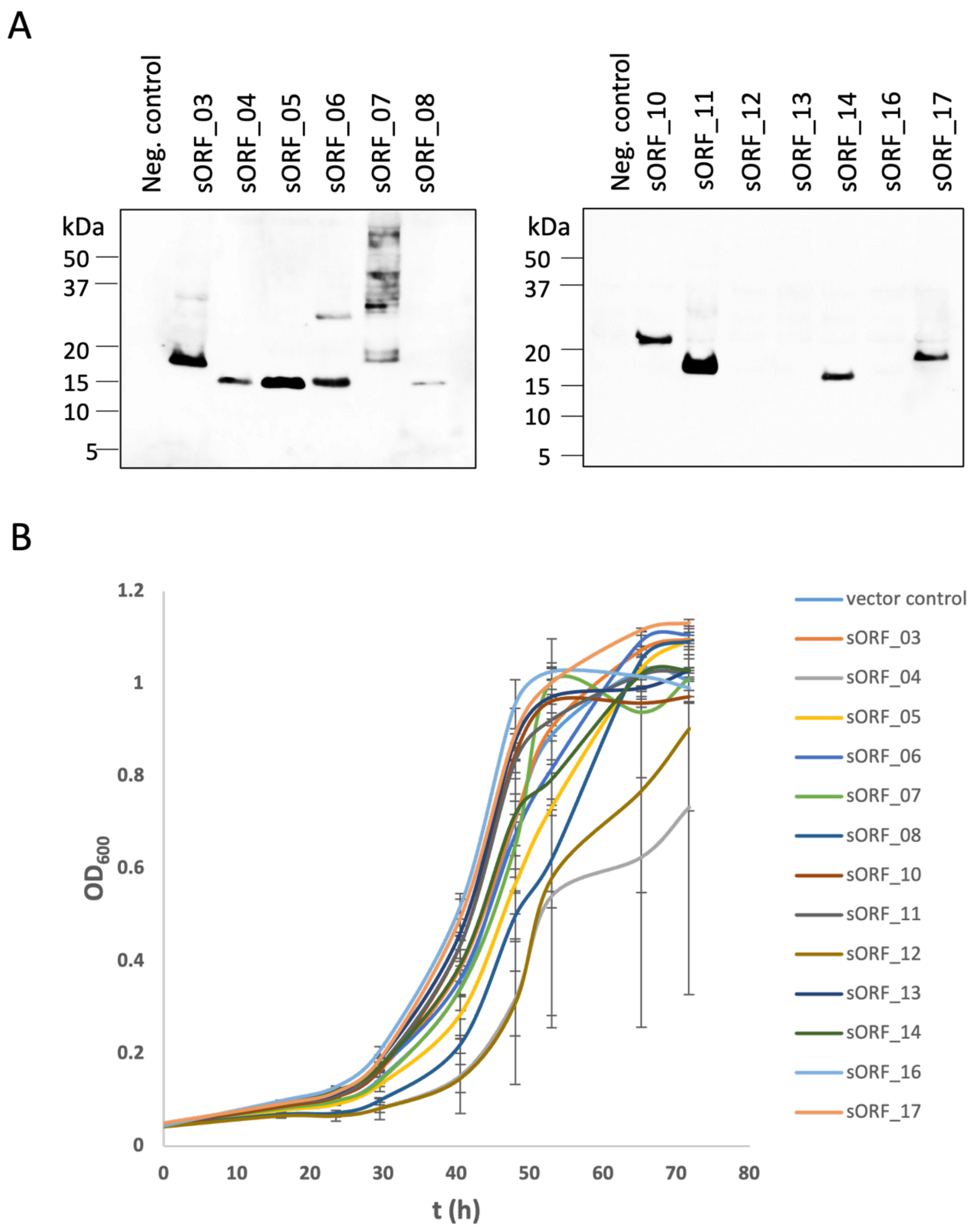

Supplementary Figure 3: Overproduction of small proteins in *M. mazei.*

(**A**) Detection of small proteins in *M. mazei* cell extract. SDS PAGE followed by western blot analysis was performed with *M. mazei* cell extract from the exponential growth phase (approx. 30 µg cell extract protein). A monoclonal FLAG-directed antibody was used to detect the SPA-tag at the C-terminus of the small protein under +N conditions with a vector based constitutive overproduction. The empty vector was used as control. All corresponding growth experiments were performed in three

biological replicates and the subsequent western blot was performed with the cell extracts from one biological replicate each. **(B)** Growth analysis of the overproduction strains of selected small proteins. Depicted are the growth curves of the overproduction strains in comparison with the *M. mazei* empty vector control under +N growth conditions with puromycin (5 µg/ml) as selection marker each in three biological replicates and standard deviation for each time point is indicated. The growth was monitored by measuring the optical density at 600 nm (OD_{600}) over the time. Source data for A and B are provided as a Source Data file.

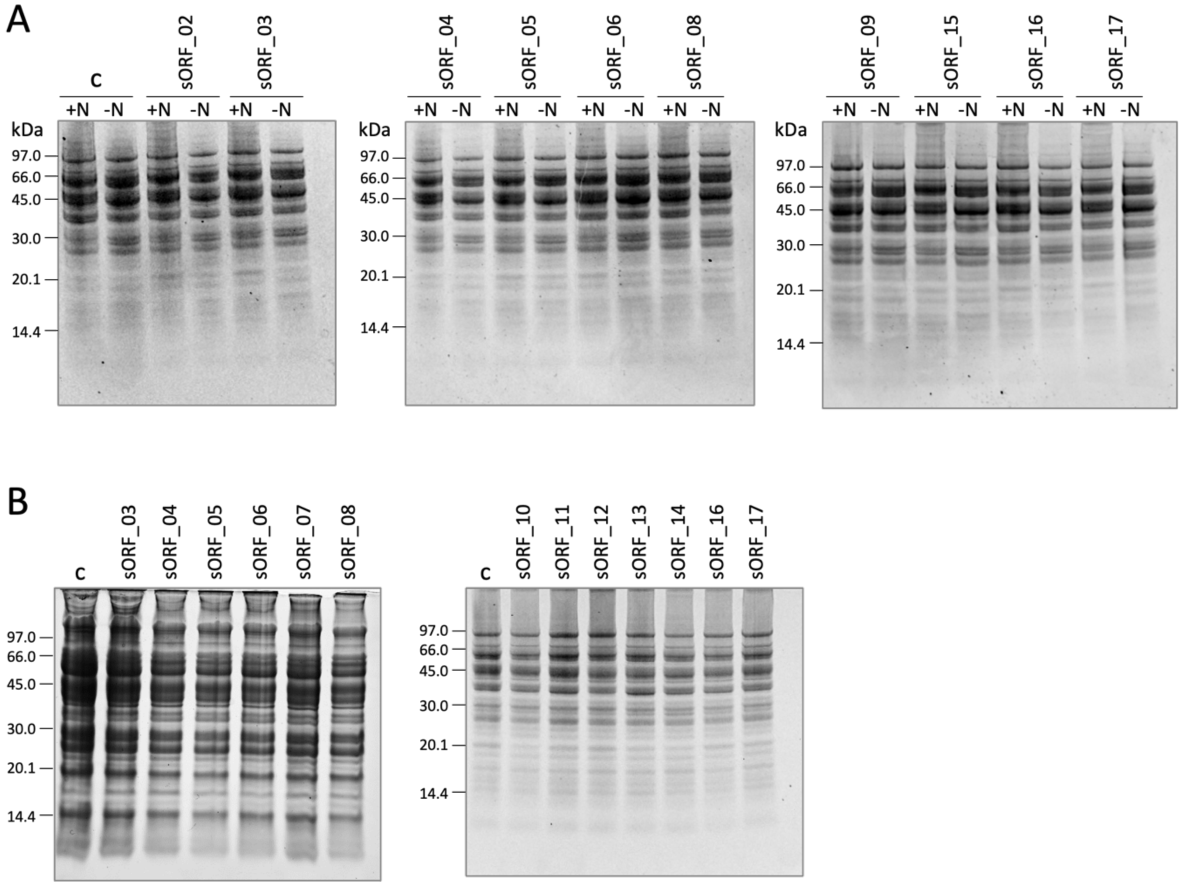

Supplementary Figure 4: Protein stained gels of *M. mazei* cell extracts as western blot controls.

SDS PAGE followed Coomassie staining was performed with 30 µg *M. mazei* cell extract from the exponential growth phase. Selected small proteins **(A)** under control of their respective native promoter and ribosome binding site were grown under +N as well as –N conditions and **(B)** under +N conditions with a vector based constitutive overproduction. The empty vector was used as control. Source data for A and B are provided as a Source Data file.

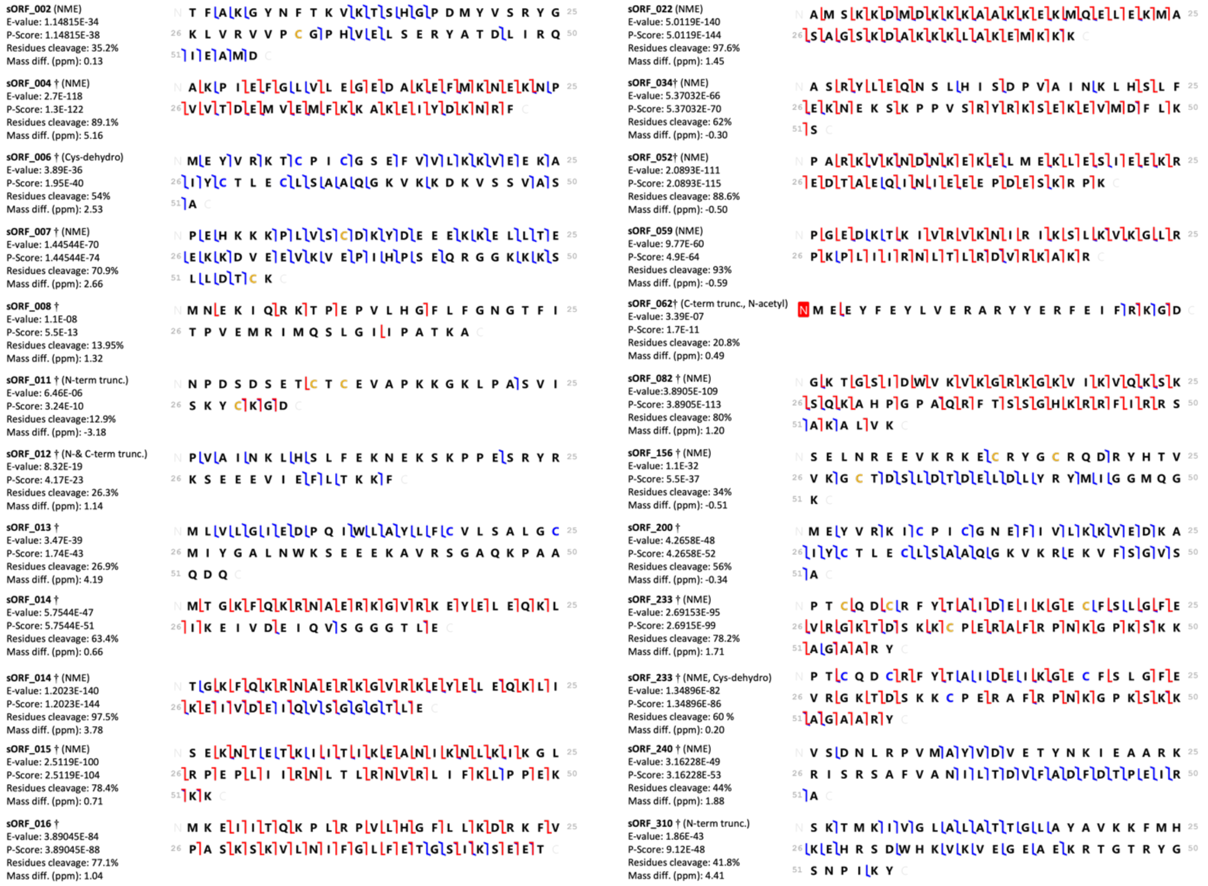

Supplementary Figure 5. Mass spectrometry-based top-down proteomics

Mass spectrometry-based top-down proteomics level evidence for the translation of sORF encoded proteins. All sORF products detected across the re-analysis of top-down proteomics datasets are presented with their associated confidence values (E-value & P-score), the percentage fragmentation achieved, and mass difference between the theoretical and expected mass (ppm). A tolerance of 10 ppm was employed at both MS and MS/MS levels. Blue lines represent b- and y-fragment ions, red lines represent c- and z-ions. NME = N-terminal methionine excision, N/C-term trunc. = N- or C-terminal truncation of the canonical amino acid sequence, Cys-dehydro = cysteine residues in the oxidised form (i.e., disulphide linkage), N-acetyl = N-terminal acetylation.

† Identified via TDP with corroborating evidence via BUP.

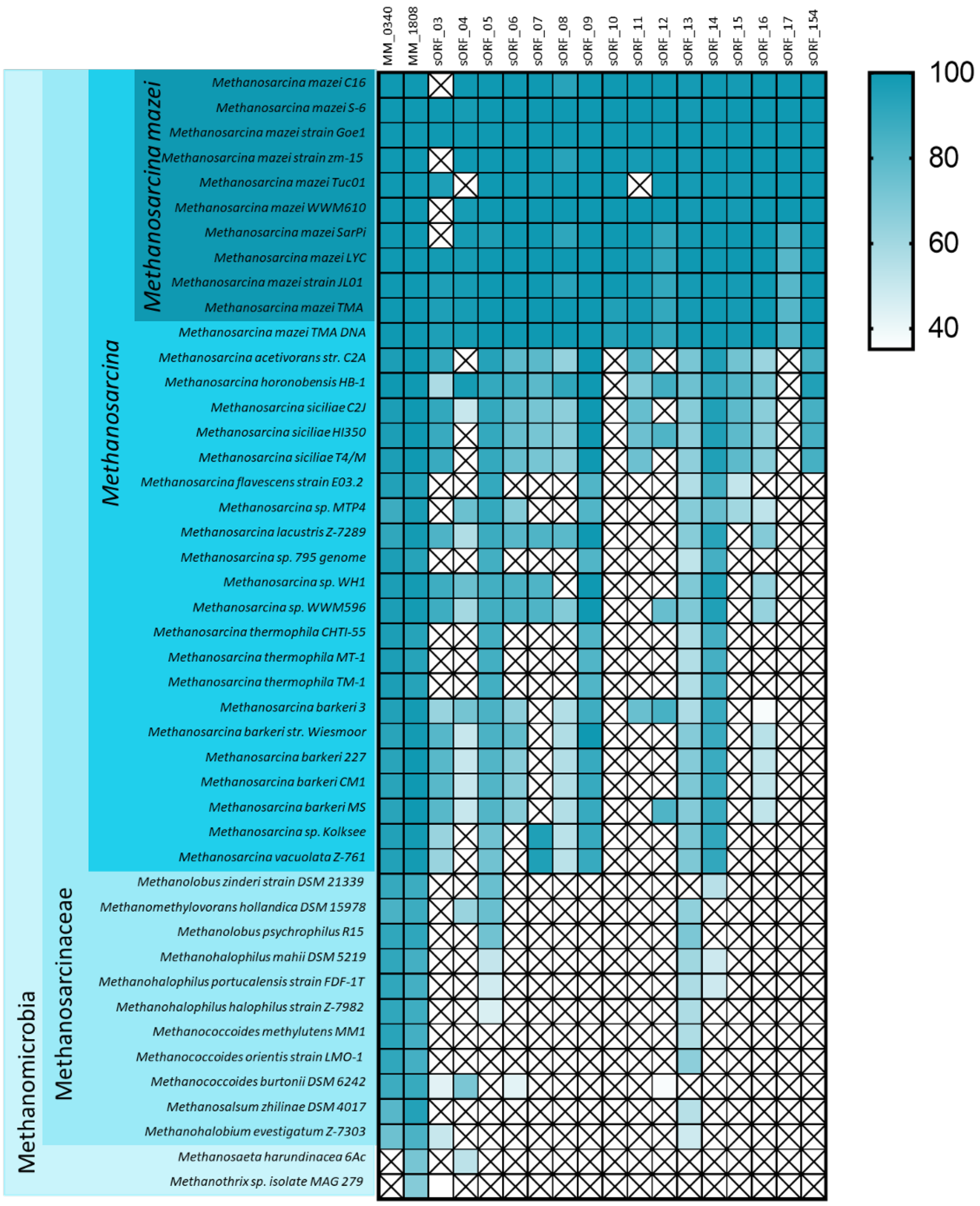

Supplementary Figure 6: Amino acid conservation of selected unannotated small proteins

The colour gradient indicates % identity of tBlastn search in Archaea. Two ribosomal proteins MM_0340 and MM_1808 are included for comparison.

A

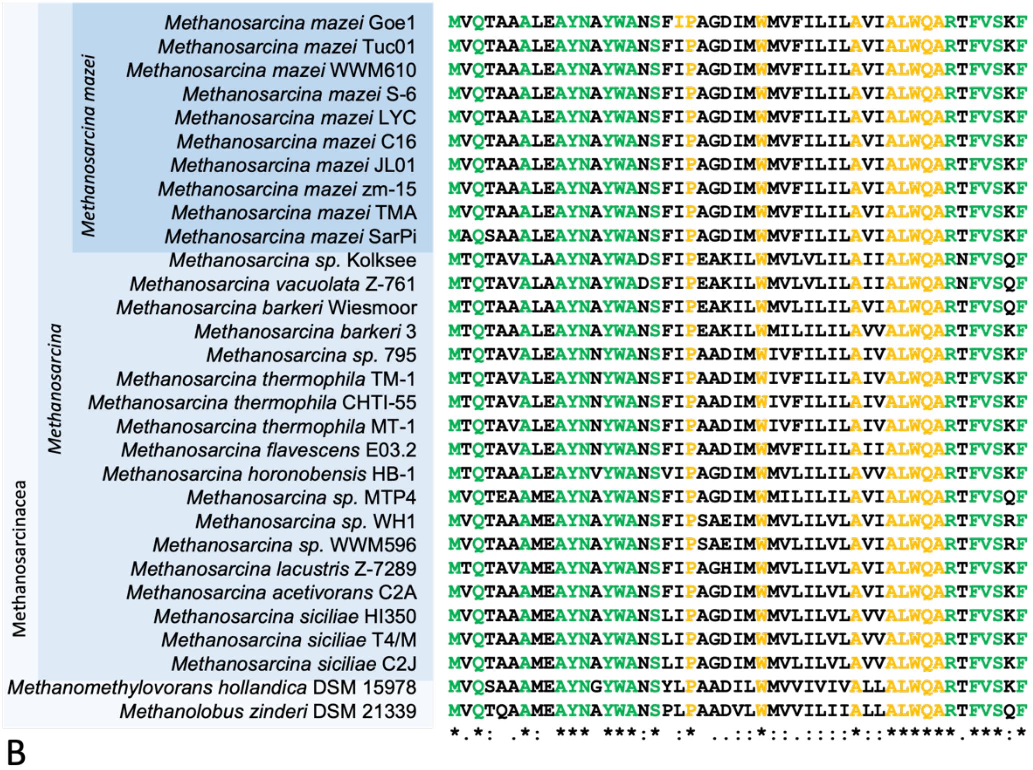
Methanosarcinacea
Methanosarcina
Methanosarcina mazei
Methanosarcina mazei Goe1 MVQTAAALEAYNAYWANSFIPAGDIMWMVFILILAVIALWQARTFVSKF
Methanosarcina mazei Tuc01 MVQTAAALEAYNAYWANSFIPAGDIMWMVFILILAVIALWQARTFVSKF
Methanosarcina mazei WWM610 MVQTAAALEAYNAYWANSFIPAGDIMWMVFILILAVIALWQARTFVSKF
Methanosarcina mazei S-6 MVQTAAALEAYNAYWANSFIPAGDIMWMVFILILAVIALWQARTFVSKF
Methanosarcina mazei LYC MVQTAAALEAYNAYWANSFIPAGDIMWMVFILILAVIALWQARTFVSKF
Methanosarcina mazei C16 MVQTAAALEAYNAYWANSFIPAGDIMWMVFILILAVIALWQARTFVSKF
Methanosarcina mazei JL01 MVQTAAALEAYNAYWANSFIPAGDIMWMVFILILAVIALWQARTFVSKF
Methanosarcina mazei zm-15 MVQTAAALEAYNAYWANSFIPAGDIMWMVFILILAVIALWQARTFVSKF
Methanosarcina mazei TMA MVQTAAALEAYNAYWANSFIPAGDIMWMVFILILAVIALWQARTFVSKF
Methanosarcina mazei SarPi MAQSAAALEAYNAYWANSFIPAGDIMWMVFILILAVIALWQARTFVSKF
Methanosarcina sp. Kolksee MTQTAVALAAYNAYWADSFIPEAKILWMVLVLILAIIALWQARNFVSQF
Methanosarcina vacuolata Z-761 MTQTAVALAAYNAYWADSFIPEAKILWMVLVLILAIIALWQARNFVSQF
Methanosarcina barkeri Wiesmoor MTQTAAALAAYNAYWANSFIPEAKILWMVLILILAVIALWQARTFVSQF
Methanosarcina barkeri 3 MTQTAAALEAYNAYWANSFIPEAKILWMILILILAVVALWQARTFVSKF
Methanosarcina sp. 795 MTQTAVALEAYNNYWANSFIPAADIMWIVFILILAIVALWQARTFVSKF
Methanosarcina thermophila TM-1 MTQTAVALEAYNNYWANSFIPAADIMWIVFILILAIVALWQARTFVSKF
Methanosarcina thermophila CHTI-55 MTQTAVALEAYNNYWANSFIPAADIMWIVFILILAIVALWQARTFVSKF
Methanosarcina thermophila MT-1 MTQTAVALEAYNNYWANSFIPAADIMWIVFILILAIVALWQARTFVSKF
Methanosarcina flavescens E03.2 MTQTAVALEAYNNYWANSFIPAADIMWMVFILILAIIALWQARTFVSKF
Methanosarcina horonobensis HB-1 MTQTAAALEAYNVYWANSVIPAGDIMWMVLILILAVVALWQARTFVSKF
Methanosarcina sp. MTP4 MVQTEAAMEAYNAYWANSFIPAGDIMWMILILILAVIALWQARTFVSQF
Methanosarcina sp. WH1 MVQTAAAMEAYNAYWANSFIPSAEIMWMVLILVLAVIALWQARTFVSRF
Methanosarcina sp. WWM596 MVQTAAAMEAYNAYWANSFIPSAEIMWMVLILVLAVIALWQARTFVSRF
Methanosarcina lacustris Z-7289 MTQTAVAMEAYNAYWANSFIPAGHIMWMVLILVLAVIALWQARTFVSKF
Methanosarcina acetivorans C2A MVQTAAAMEAYNAYWANSFIPAGDIMWMVLILVLAVIALWQARTFVSKF
Methanosarcina siciliae HI350 MVQTAAAMEAYNAYWANSLIPAGDIMWMVLILVLAVVALWQARTFVSKF
Methanosarcina siciliae T4/M MVQTAAAMEAYNAYWANSLIPAGDIMWMVLILVLAVVALWQARTFVSKF
Methanosarcina siciliae C2J MVQTAAAMEAYNAYWANSLIPAGDIMWMVLILVLAVVALWQARTFVSKF
Methanomethylovorans hollandica DSM 15978 MVQSAAAMEAYNGYWANSYLPAADILWMVVIVIVALLALWQARTFVSKF
Methanolobus zinderi DSM 21339 MVQTQAAMEAYNAYWANSPLPAADVLWMVVILIIALLALWQARTFVSQF
.: .*: *** ***:* :* ..::*::.::::*::******.***:*

B

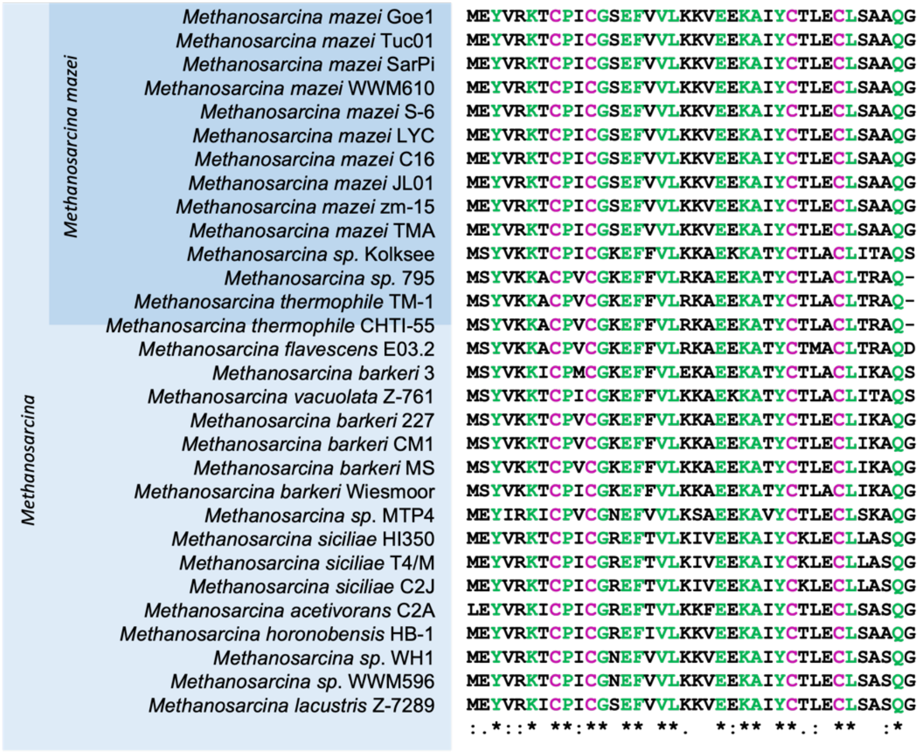
Methanosarcina
Methanosarcina mazei
Methanosarcina mazei Goe1 MEYVRKTCPICGSEFVVLKKVEEKAIYCTLECLSAAQG
Methanosarcina mazei Tuc01 MEYVRKTCPICGSEFVVLKKVEEKAIYCTLECLSAAQG
Methanosarcina mazei SarPi MEYVRKTCPICGSEFVVLKKVEEKAIYCTLECLSAAQG
Methanosarcina mazei WWM610 MEYVRKTCPICGSEFVVLKKVEEKAIYCTLECLSAAQG
Methanosarcina mazei S-6 MEYVRKTCPICGSEFVVLKKVEEKAIYCTLECLSAAQG
Methanosarcina mazei LYC MEYVRKTCPICGSEFVVLKKVEEKAIYCTLECLSAAQG
Methanosarcina mazei C16 MEYVRKTCPICGSEFVVLKKVEEKAIYCTLECLSAAQG
Methanosarcina mazei JL01 MEYVRKTCPICGSEFVVLKKVEEKAIYCTLECLSAAQG
Methanosarcina mazei zm-15 MEYVRKTCPICGSEFVVLKKVEEKAIYCTLECLSAAQG
Methanosarcina mazei TMA MEYVRKTCPICGSEFVVLKKVEEKAIYCTLECLSAAQG
Methanosarcina sp. Kolksee MSYVKKTCPICGKEFFVLKKAEKKATYCTLACLITAQS
Methanosarcina sp. 795 MSYVKKACPVCGKEFFVLRKAEEKATYCTLACLTRAQ-
Methanosarcina thermophile TM-1 MSYVKKACPVCGKEFFVLRKAEEKATYCTLACLTRAQ-
Methanosarcina thermophile CHTI-55 MSYVKKACPVCGKEFFVLRKAEEKATYCTLACLTRAQ-
Methanosarcina flavescens E03.2 MSYVKKACPVCGKEFFVLRKAEEKATYCTMACLTRAQD
Methanosarcina barkeri 3 MSYVKKICPMCGKEFFVLEKAEEKATYCTLACLIKAQS
Methanosarcina vacuolata Z-761 MSYVKKTCPICGKEFFVLKKAEKKATYCTLACLITAQS
Methanosarcina barkeri 227 MSYVKKTCPVCGKEFFVLKKAEEKATYCTLECLIKAQG
Methanosarcina barkeri CM1 MSYVKKTCPVCGKEFFVLKKAEEKATYCTLECLIKAQG
Methanosarcina barkeri MS MSYVKKTCPVCGKEFFVLKKAEEKATYCTLECLIKAQG
Methanosarcina barkeri Wiesmoor MSYVKKTCPICGKEFFVLKKAEEKATYCTLACLIKAQG
Methanosarcina sp. MTP4 MEYIRKICPVCGNEFVVLKSAEEKAVYCTLECLSKAQG
Methanosarcina siciliae HI350 MEYVRKTCPICGREFTVLKIVEEKAIYCKLECLLASQG
Methanosarcina siciliae T4/M MEYVRKTCPICGREFTVLKIVEEKAIYCKLECLLASQG
Methanosarcina siciliae C2J MEYVRKTCPICGREFTVLKIVEEKAIYCKLECLLASQG
Methanosarcina acetivorans C2A LEYVRKICPICGREFTVLKKFEEKAIYCTLECLSASQG
Methanosarcina horonobensis HB-1 MEYVRKTCPICGREFIVLKKVEEKAIYCTLECLSAAQG
Methanosarcina sp. WH1 MEYVRKTCPICGNEFVVLKKVEEKAIYCTLECLSASQG
Methanosarcina sp. WWM596 MEYVRKTCPICGNEFVVLKKVEEKAIYCTLECLSASQG
Methanosarcina lacustris Z-7289 MEYVRKICPICGSEFVVLKKVEEKAIYCTLECLSASQG
:.*::* **:** ** **. *:** **.: ** :*

Supplementary Figure 7: Amino acid alignments of small proteins encoded by sORF_05 and sORF_06

Conserved amino acid sequences from tBlastn analysis were aligned using ClustalOmega. **(A)** Alignment of small protein encoded by sORF_05 and homologs, conserved amino acids are labelled in green and in yellow inside the predicted α-helix. **(B)** Alignment of homologs of sORF_06 encoded small protein, conserved amino acids are labelled in green, conserved cysteines are visualized in pink.

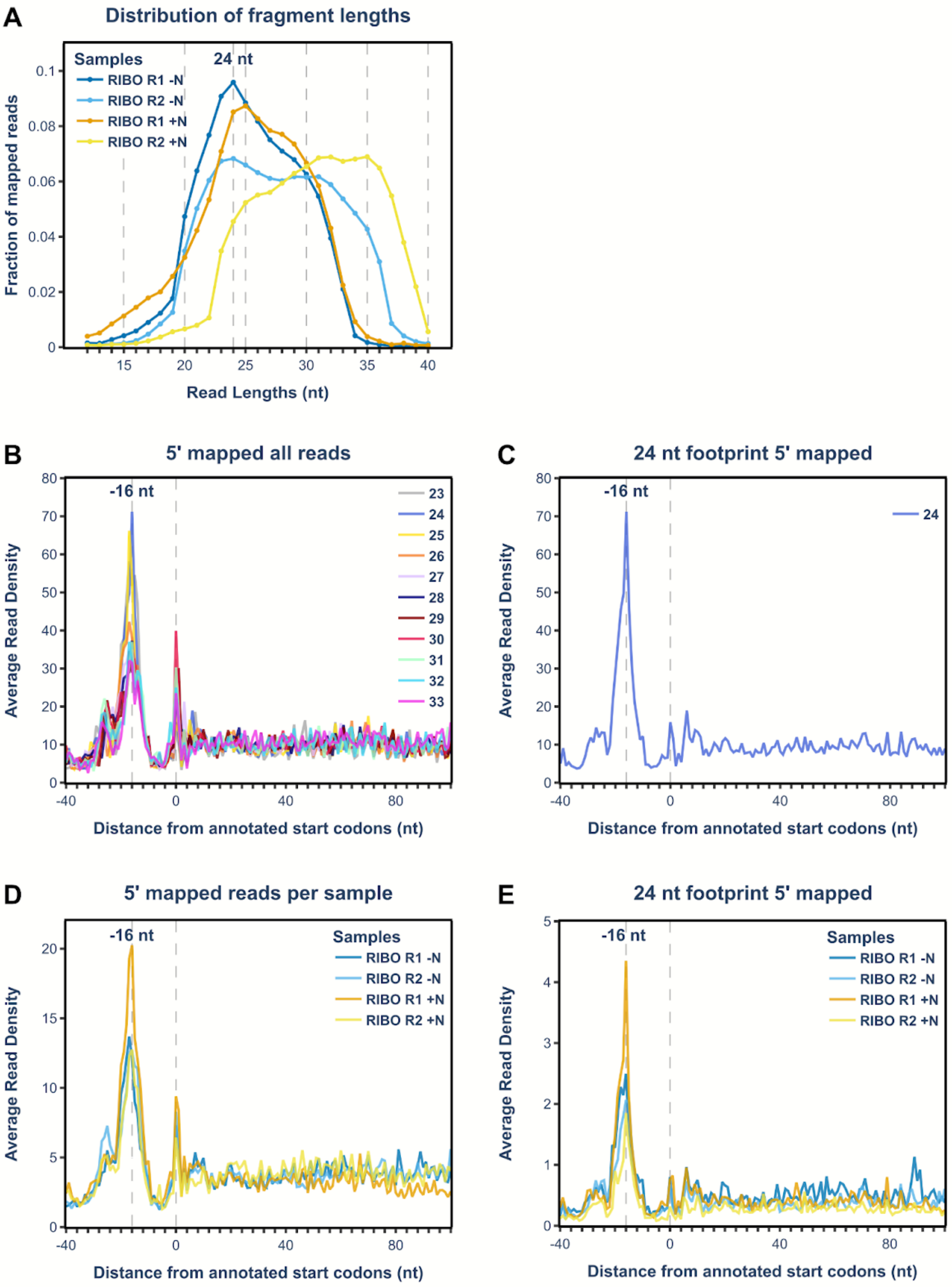

A
Distribution of fragment lengths
Samples
RIBO R1 -N
RIBO R2 -N
RIBO R1 +N
RIBO R2 +N
24 nt
Fraction of mapped reads
Read Lengths (nt)
B
5' mapped all reads
-16 nt
23
24
25
26
27
28
29
30
31
32
33
Average Read Density
Distance from annotated start codons (nt)
C
24 nt footprint 5' mapped
-16 nt
24
Average Read Density
Distance from annotated start codons (nt)
D
5' mapped reads per sample
-16 nt
Samples
RIBO R1 -N
RIBO R2 -N
RIBO R1 +N
RIBO R2 +N
Average Read Density
Distance from annotated start codons (nt)
E
24 nt footprint 5' mapped
-16 nt
Samples
RIBO R1 -N
RIBO R2 -N
RIBO R1 +N
RIBO R2 +N
Average Read Density
Distance from annotated start codons (nt)

Supplementary Figure 8: Distribution of Fragment Lengths and Read Density Around Annotated Start Codons.

(A) Distribution of footprint fragment lengths for each replicate/library, showing a predominance of 23-25nt long fragments. **(B)** Density profiles of 5'-mapped reads around annotated start codons for distinct read lengths in the range of 23-33nt, with reads from all libraries combined. **(C)** Density profiles of 5'-mapped reads around annotated start codons specifically for the most abundant read length of 24nt, with reads from all libraries combined. **(D)** Density profiles of 5'-mapped reads around annotated start codons for each library, including all read lengths in the 23-33nt range. **(E)** Density profiles of 5'-mapped reads around annotated start codons for each library, specifically focusing on the most abundant read length of 24nt. (B-E) All profiles demonstrate the highest read density occurring 16nt upstream of the start codon, with a noticeable shift towards position 0 for longer read lengths.

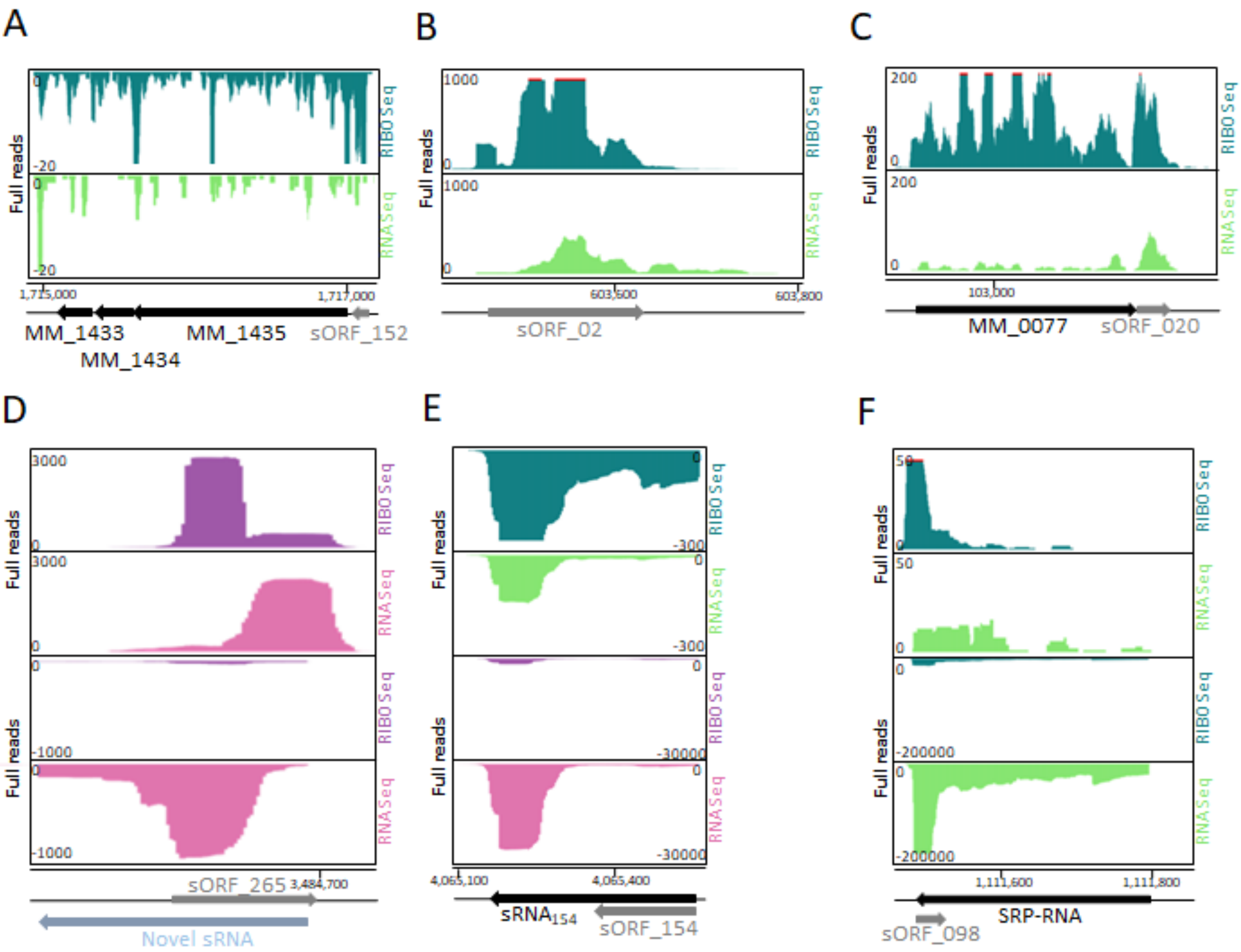

Supplementary Figure 9: Unannotated sORFs are found in different genomic regions

Examples are visualized as screenshot from the genome browser. 5'UTR **(A)**, intergenic **(B)**, in an operon with a long ORF **(C)**, potential toxin-antitoxin system **(D)**, within an sRNA **(E)**, and antisense to a long ORF **(F)**. Green is +N condition, pink is -N condition.

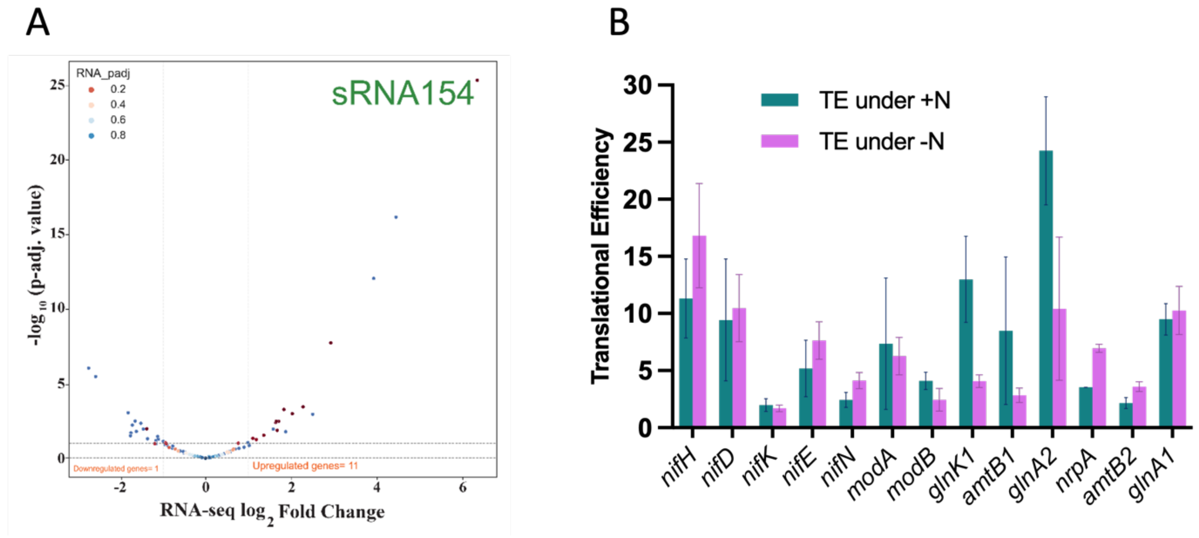

Supplementary Figure 10: Differential expression under -N vs. +N conditions

(A) Volcano plot of regulation of small RNA on transcript level, highlighted is sRNA154. **(B)** Translation efficiency of selected genes of interest under +N (green) and –N (pink). Bars showing the Mean ± SD translational efficiency (TE = Ribo-seq/RNA-seq) computed from two independent experiments with two replicates in each experiment for both RIBOseq and RNAseq libraries.

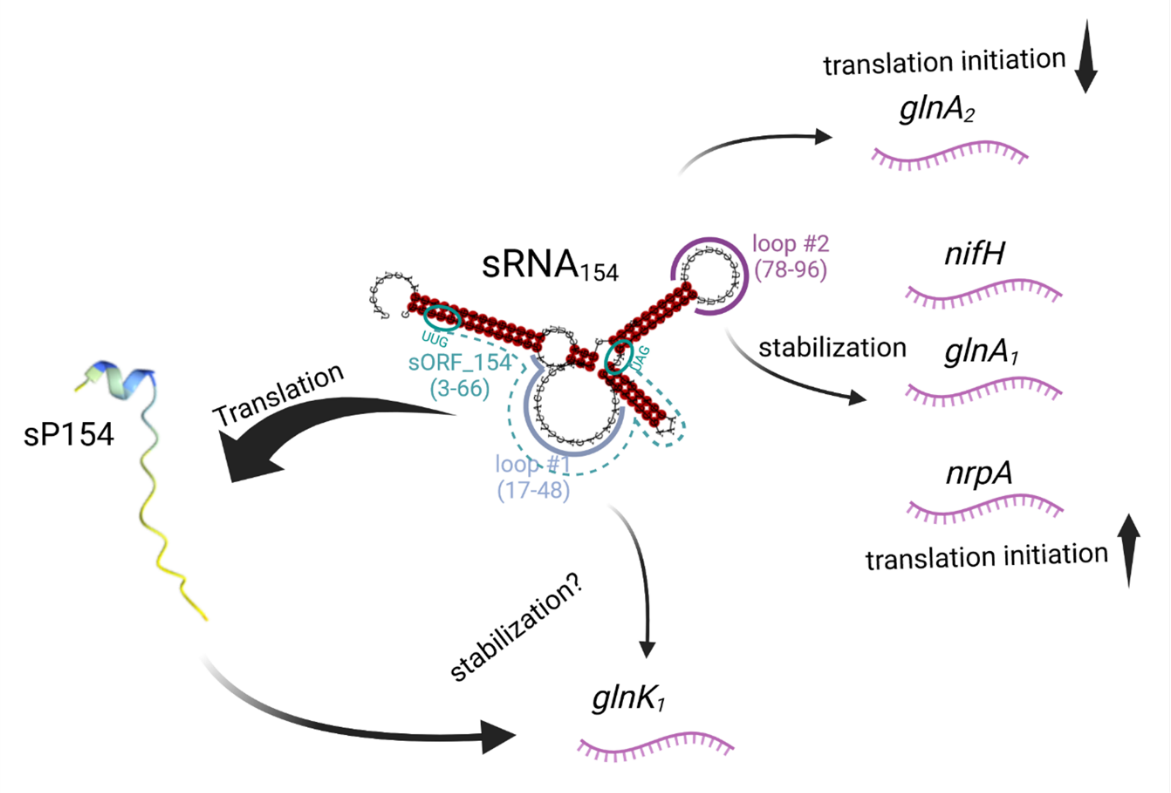

Supplementary Figure 11: Model of posttranscriptional regulation by sRNA$_{154}$

sRNA$_{154}$ loop #2 (purple) stabilizes the mRNA of *nifH* and *glnA$_1$* and upregulates the translation initiation of *nrpA*. The translation initiation of *glnA$_2$* is downregulated by loop #2 [1]. The predicted sORF_154 location is visualized in turquois, dotted line, start and stop codon are highlighted with a circle. The small protein sP154 might stabilize the mRNA of *glnK$_1$* either in combination with loop #1 or by direct interaction with *glnK$_1$* mRNA. The model was partially created with https://www.biorender.com/ released under a Creative Commons Attribution-NonCommercial-NoDerivs 4.0 International license (https://creativecommons.org/licenses/by-nc-nd/4.0/deed.en).

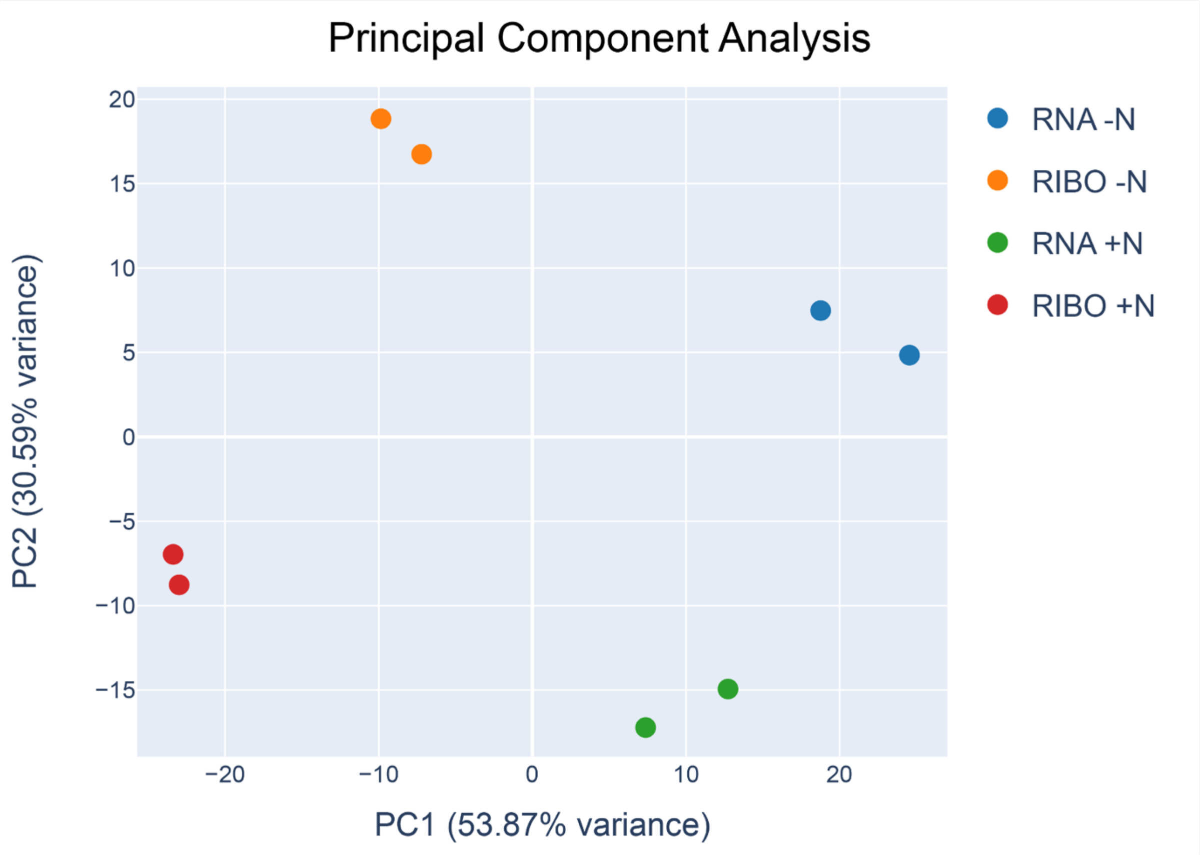

Supplementary Figure 12: Principal Component Analysis (PCA)

PCA was applied to median-of-ratios normalized and regularized log-transformed (rlog) read count values from *Methanosarcina mazei* Ribo-seq (RIBO) and RNA-seq (RNA) datasets. These datasets were obtained under Nitrogen-depleted (-N) and Nitrogen-rich (+N) conditions. The x-axis of the plot represents the first principal component, which accounts for the largest proportion of variance in the data (53.87%). The y-axis represents the second principal component, accounting for 30.59% of the variance. Notably, the plot reveals that the replicates of each respective method align closely, following the same trend.

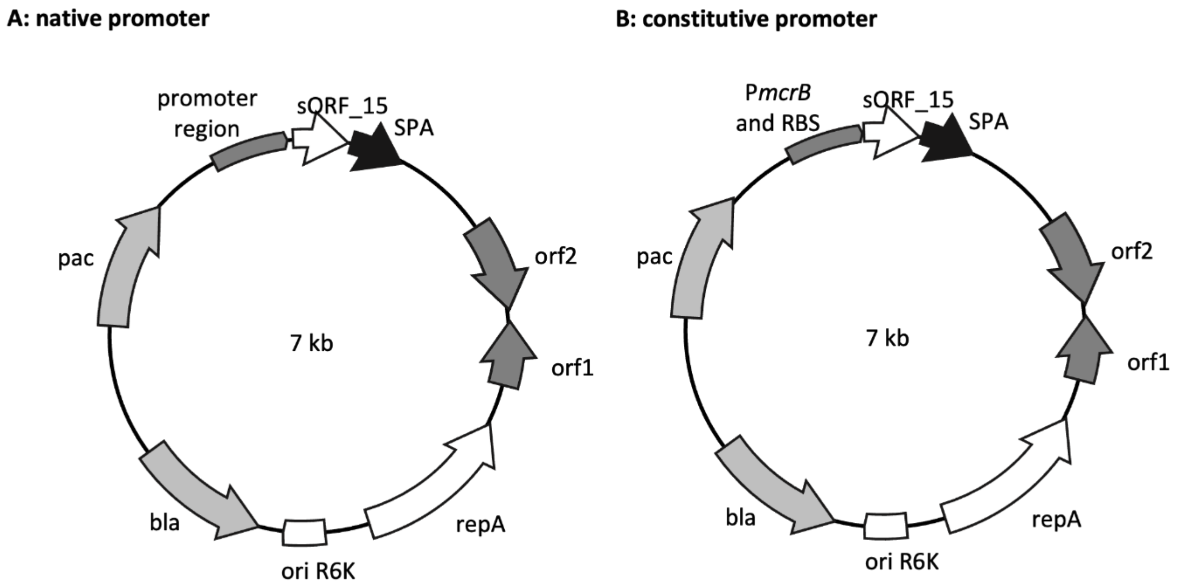

Supplementary Figure 13: Plasmid maps exemplarily shown for sORF_15

The sORF was C-terminally fused to SPA-tag under the control of the native promoter region **(A)** containing approximately 200 nt upstream of the predicted start of translation and **(B)** under the control of the constitutive promoter P*mcrB* for constant expression in pRS1595.

1. Supplementary Tables:

Supplementary Table 1: List of strains used in this study

Organism	Description	Reference
Escherichia coli DH5α	general cloning strain	Hanahan 1938 [2]
E. coli DH5α λpir	general cloning strain	Miller and Melakanos 1988 [3]
Methanosarcina mazei Gö1	wildtype	DSMZ No. 3647
*M. mazei**	potential cell wall mutant	Ehlers et al., 2005 [4]
M. mazei mut 281	riboORF_03 under PmcrB with C-term. SPA-tag	this study
M. mazei mut 264	riboORF_04 under PmcrB with C-term. SPA-tag	this study
M. mazei mut 265	riboORF_05 under PmcrB with C-term. SPA-tag	this study
M. mazei mut 269	riboORF_06 under PmcrB with C-term. SPA-tag	this study
M. mazei mut 279	riboORF_07 under PmcrB with C-term. SPA-tag	this study
M. mazei mut 266	riboORF_08 under PmcrB with C-term. SPA-tag	this study
M. mazei mut 282	riboORF_10 under PmcrB with C-term. SPA-tag	this study
M. mazei mut 296	riboORF_11 under PmcrB with C-term. SPA-tag	this study
M. mazei mut 267	riboORF_12 under PmcrB with C-term. SPA-tag	this study
M. mazei mut 286	riboORF_13 under PmcrB with C-term. SPA-tag	this study
M. mazei mut 278	riboORF_14 under PmcrB with C-term. SPA-tag	this study
M. mazei mut 287	riboORF_16 under PmcrB with C-term. SPA-tag	this study
M. mazei mut 268	riboORF_17 under PmcrB with C-term. SPA-tag	this study
M. mazei mut 361	riboORF_02 under nativem Promotor with C-term. SPA-tag	this study
M. mazei mut 362	riboORF_03 under nativem Promotor with C-term. SPA-tag	this study
M. mazei mut 363	riboORF_04 under nativem Promotor with C-term. SPA-tag	this study
M. mazei mut 364	riboORF_05 under nativem Promotor with C-term. SPA-tag	this study
M. mazei mut 365	riboORF_06 under nativem Promotor with C-term. SPA-tag	this study
M. mazei mut 366	riboORF_07 under nativem Promotor with C-term. SPA-tag	this study
M. mazei mut 260	riboORF_08 under nativem Promotor with C-term. SPA-tag	this study
M. mazei mut 284	riboORF_09 under nativem Promotor with C-term. SPA-tag	this study
M. mazei mut 259	riboORF_15 under nativem Promotor with C-term. SPA-tag	this study
M. mazei mut 367	riboORF_16 under nativem Promotor with C-term. SPA-tag	this study
M. mazei mut 368	riboORF_17 under nativem Promotor with C-term. SPA-tag	this study

Supplementary Table 2: List of plasmids used in this study

Plasmid	Description	Reference
pBSK+	cloning vector	Statagene
pCRII TOPO	cloning vector	Invitrogen
pLH7.3	contains SPA-tag	Group Sharma, unpublished
pRS1595	shuttle vector	Thomsen et al., 2022 [5]
pRS1598	sORF_03 in pRS893	this study
pRS1599	sORF_04 in pRS893	this study
pRS1600	sORF_05 in pRS893	this study
pRS1601	sORF_07 in pRS893	this study
pRS1602	sORF_08 in pRS893	this study
pRS1603	sORF_09 in pRS893	this study
pRS1604	sORF_13 in pRS893	this study
pRS1606	sORF_16 in pRS893	this study
pRS1607	sORF_17 in pRS893	this study
pRS1609	sORF_06 in pRS893	this study
pRS1610	sORF_12 in pRS893	this study
pRS1611	sORF_14 in pRS893	this study
pRS1612	SPA in pCRII TOPO	this study
pRS1624	SPA in pRS1595	this study
pRS1627	pRS1598 Δ NcoI restriction site	this study
pRS1628	pRS1600 Δ NcoI restriction site	this study
pRS1629	pRS1601 Δ NcoI restriction site	this study
pRS1630	pRS1602 Δ NcoI restriction site	this study
pRS1631	pRS1603 Δ NcoI restriction site	this study
pRS1632	pRS1606 Δ NcoI restriction site	this study
pRS1634	pRS1609 Δ NcoI restriction site	this study
pRS1635	pRS1610 Δ NcoI restriction site	this study
pRS1636	pRS1611 Δ NcoI restriction site	this study
pRS1637	native promotor-sORF_08-SPA in pRS1595	this study
pRS1638	native promotor-sORF_15-SPA in pRS1595	this study
pRS1639	native promotor-sORF_16-SPA in pRS1595	this study
pRS1648	pRS1599 Δ NcoI restriction site	this study
pRS1650	pRS1607 Δ NcoI restriction site	this study
pRS1651	pmcrB-sORF_05-SPA in pRS1595	this study
pRS1655	pmcrB-sORF_06-SPA in pRS1595	this study
pRS1657	pmcrB-sORF_03-SPA in pRS1595	this study
pRS1658	pmcrB-sORF_04-SPA in pRS1595	this study
pRS1659	pmcrB-sORF_08-SPA in pRS1595	this study
pRS1660	pmcrB-sORF_09-SPA in pRS1595	this study
pRS1661	pmcrB-sORF_12-SPA in pRS1595	this study
pRS1662	pmcrB-sORF_14-SPA in pRS1595	this study

pRS1663	pmcrB-sORF_17-SPA in pRS1595	this study
pRS1664	sORF_10 in pRS893	this study
pRS1668	pmcrB-sORF_07-SPA in pRS1595	this study
pRS1670	pmcrB-sORF_16-SPA in pRS1595	this study
pRS1673	pRS1604 Δ NcoI restriction site	this study
pRS1674	native promotor-sORF_06 in pCRII TOPO	this study
pRS1679	pRS1664 Δ NcoI restriction site	this study
pRS1684	native promotor-sORF_09 in pCRII TOPO	this study
pRS1685	sORF_11 in pCRII TOPO	this study
pRS1689	native promotor-sORF_06-SPA in pRS1595	this study
pRS1690	native promotor-sORF_09-SPA in pRS1595	this study
pRS1691	pmcrB-sORF_10-SPA in pRS1595	this study
pRS1692	sORF_11 in pRS893	this study
pRS1700	pmcrB-sORF_13-SPA in pRS1595	this study
pRS1701	pRS1700 Δ NcoI restriction site	this study
pRS1702	pRS1692 Δ NcoI restriction site	this study
pRS1704	pmcrB-sORF_11-SPA in pRS1595	this study
pRS893	pmcrB + M. mazei RBS in pDrive	Group Schmitz-streit, unpublished
pRS20238	native promotor-sORF_02-SPA in pRS1595	this study
pRS20239	native promotor-sORF_03-SPA in pRS1595	this study
pRS20240	native promotor-sORF_04-SPA in pRS1595	this study
pRS20241	native promotor-sORF_05-SPA in pRS1595	this study
pRS20242	native promotor-sORF_07-SPA in pRS1595	this study
pRS20244	native promotor-sORF_017-SPA in pRS1595	this study

Supplementary Table 3: List of Oligonucleotides used in this study

Primer	Sequence 5'-3'
pmcrB sd new	TTTAATTTCCTCCTTAATTTATTAAAATCACTTTGGGACTGGTCACCTAATCGAGTG
PriboORF_02 XhoI for	TAGACTCGAGATGAAGCCTGTTAACTGA
riboORF_02 BamHI rev	TAGAGGATCCATCCATGGCTTCGATCTGGC
riboORF_03 BamHI rev	TAGAGGATCCTTTCCTGACCATAGGGCTTCCG
riboORF_03 NcoI for	TAGACCATGGATGCCAAGAGGAGACAGAACAGGTC
PriboORF_03 XhoI for	TAGACTCGAGTTTCCGAAGAGATTTTGTT
riboORF_04 BamHI rev	TAGAGGATCCAAACCTATTTTTATCATATATCTCTTTA
riboORF_04 NcoI for	TAGACCATGGATGGCGAAACCTATTGAGTT
PriboORF_04 XhoI for	TAGACTCGAGAATCTTTTTTAAATAA
riboORF_05 BamHI rev	TAGAGGATCCGAATTTGGACACGAAAGTCCT
riboORF_05 NcoI for	TAGACCATGGATGGTACAAACAGCAGCAGCT
PriboORF_05 XhoI for	TAGACTCGAGAACAGTGCATACGGAA
PriboORF_06 XhoI for	TAGACTCGAGGGAAGAAATATCAAAATGAAAGTGAG
riboORF_06 BamHI rev	TAGAGGATCCTGCAGACGCCACGGAAGAAACT
riboORF_06 NcoI for	TAGACCATGGATGGAGTATGTAAGAAAAACCTGTCCA
riboORF_07 BamHI rev	TAGAGGATCCCTTGCACGTGTCAAGA
riboORF_07 NcoI for	TAGACCATGGATGCCAGAACATAAGAAAAAG
PriboORF_07 XhoI for	TAGACTCGAGAATACTGTTCAGGAGA
PriboORF_08 XhoI for	TAGACTCGAGCTTGACCTGGAGCTTTGAC
riboORF_08 BamHI rev	TAGAGGATCCTGCTTTTGTTGCCGGAATGA
riboORF_08 NcoI for	TAGACCATGGATGAACGAAAAAATACAGAGA
PriboORF_09 XhoI for 2	TAGACTCGAGTTGGGCATTTTCAGATT
riboORF_09 BamHI rev	TAGAGGATCCCATATTCGGTACTGAAACTTTTAT
riboORF_09 NcoI for	TAGACCATGGATGGAAGAGATATTCAAAGGATTGG
riboORF_10 BamHI rev	TAGAGGATCCCAGCCTTGCCGTTTTGAATGAA
riboORF_10 NcoI for	TAGACCATGGATGGGAGCAATGAGGGAGGAAGCAG
riboORF_11 BamHI rev	TAGAGGATCCGGGCATCATGTGTGAACGG
riboORF_11 NcoI for	TAGACCATGGGTGTATTGCCGTTATCTCATA
riboORF_12 BamHI rev	TAGAGGATCCAAATTTTTTAGTTAAGAATTCGATTAC
riboORF_12 NcoI for	TAGACCATGGATGGCGTCGAGATATCTGGAA
riboORF_13 NcoI for	TAGACCATGGATGTTAGTTTTAGGAATTGAGG
riboORF_13 NcoI rev	TAGACCATGGCCTAGGTTGATCCTGAGCTGCTG
riboORF_13 sd	ATGTTAGTTTTAGGAATTGAGGATCC
riboORF_14 BamHI rev	TAGAGGATCCCTCTAGTGTTCCACCGC
riboORF_14 NcoI for	TAGACCATGGATGACAGGAAAGTTCCAGAA
PriboORF_15 XhoI for	TAGACTCGAGGACGCTTTCGGAAAGGATAG
riboORF_15 BamHI rev	TAGAGGATCCTTTCTTCTTTTCTGGCGGGA
PriboORF_16 XhoI for	TAGACTCGAGTGGAGTGGTCTATGTCTGGGTA
riboORF_16 BamHI rev	TAGAGGATCCGGTCTCCTCCGACTTTATGCTT
riboORF_16 NcoI for	TAGACCATGGATGAAAGAAATTATTACGCAAAAGCCTCTGAGAC

riboORF_17 BamHI rev	TAGAGGATCCACCTTGCAAGGGTTCTTGGAGA
riboORF_17 NcoI for	TAGACCATGGATGAGTAAGAACTGGACGGAGAGGA
PriboORF_17 XhoI for	TAGACTCGAGAAGGAAAGGGTGAA
SPA BamHI for	TAGAGGATCCGCGGAAAAGAGAAGATG
SPA NotI rev	GCGGCCGCCTACTTGTCATCGTCATCCT

Supplementary References

1 Prasse, D., Förstner, K. U., Jäger, D., Backofen, R. & Schmitz, R. A. sRNA154 a newly identified regulator of nitrogen fixation in *Methanosarcina mazei* strain Gö1. *RNA Biology* **14**, 1544-1558 (2017). https://doi.org/10.1080/15476286.2017.1306170

2 Hanahan, D. Studies on transformation of Escherichia coli with plasmids. *J Mol Biol* **166**, 557-580 (1983). https://doi.org/https://doi.org/10.1016/S0022-2836(83)80284-8

3 Miller, V. L. & Mekalanos, J. J. A novel suicide vector and its use in construction of insertion mutations: osmoregulation of outer membrane proteins and virulence determinants in Vibrio cholerae requires toxR. *Journal of Bacteriology* **170**, 2575-2583 (1988). https://doi.org/10.1128/jb.170.6.2575-2583.1988

4 Ehlers, C. *et al.* Development of genetic methods and construction of a chromosomal *glnK1* mutant in *Methanosarcina mazei* strain Gö1. *Mol Genet Genomics* **273**, 290-298 (2005). https://doi.org/10.1007/s00438-005-1128-7

5 Thomsen, J. & Schmitz, R. A. Generating a Small Shuttle Vector for Effective Genetic Engineering of Methanosarcina mazei Allowed First Insights in Plasmid Replication Mechanism in the Methanoarchaeon. *Int J Mol Sci* **23** (2022). https://doi.org/10.3390/ijms231911910
