# Supplementary material for: Uncovering the small proteome of Methanosarcina mazei using Ribo-seq and peptidomics under different nitrogen conditions
Source: Nat Commun. 2024 Oct 6;15:8659. doi: 10.1038/s41467-024-53008-8 (PMC11456600; doi:10.1038/s41467-024-53008-8)
Supplement: Supplementary file 3 — Description of additional supplementary files [file 41467_2024_53008_MOESM3_ESM.pdf]

Description of Additional Supplementary Files

Supplementary Data 1: List of unannotated long ORFs that were not previously annotated in the annotation version used in this analysis

Supplementary Data 2: List of differentially expressed annotated ORFs found in M. mazei under -N vs. +N

Supplementary Data 3: List of 184 annotated sORFs predicted by DeepRibo, 93 of which are translated under +N, 95 under -N, and 92 under both -N and +N

Supplementary Data 4: Overview table from HRIBO output after Ribo-seq Analysis

Supplementary Data 5: List of 314 unannotated sORFs predicted by DeepRibo or NCBI ORF finder tool and manual confirmation of translation

Supplementary Data 6: Top-down proteomics evidence for translation of sORF products with the various proteoforms identified for each sORF

Supplementary Data 7: Botttom -up proteomics evidence for translation of sORF products identified for the sORF products that were either not detected via TDP, or detected via TDP with less than 30% residue cleavage.

Supplementary Data 8: List of upstream ORFs (Data Extracted from Supplementary Data 5)

Supplementary Data 9: List of Dual function sRNAs

Supplementary Data 10: Meta-data for LC-MS RAW data submission
